# Current Understanding of the “Insight” Phenomenon Across Disciplines

**DOI:** 10.3389/fpsyg.2021.791398

**Published:** 2021-12-15

**Authors:** Antonio J. Osuna-Mascaró, Alice M. I. Auersperg

**Affiliations:** Messerli Research Institute, University of Veterinary Medicine, Medical University of Vienna, University of Vienna, Vienna, Austria

**Keywords:** insight, comparative cognition, problem solving, neuroimaging, comparative psychology

## Abstract

Despite countless anecdotes and the historical significance of insight as a problem solving mechanism, its nature has long remained elusive. The conscious experience of insight is notoriously difficult to trace in non-verbal animals. Although studying insight has presented a significant challenge even to neurobiology and psychology, human neuroimaging studies have cleared the theoretical landscape, as they have begun to reveal the underlying mechanisms. The study of insight in non-human animals has, in contrast, remained limited to innovative adjustments to experimental designs within the classical approach of judging cognitive processes in animals, based on task performance. This leaves no apparent possibility of ending debates from different interpretations emerging from conflicting schools of thought. We believe that comparative cognition has thus much to gain by embracing advances from neuroscience and human cognitive psychology. We will review literature on insight (mainly human) and discuss the consequences of these findings to comparative cognition.

## Introduction

A 7years old girl is standing at a table into which psychologists have fixed a vertical transparent tube containing a small basket with a handle and a sparkly sticker inside. On the table, alongside the tubes, lie a long straight piece of pipe-cleaner and a colorful string. After inserting her finger which only reaches down about a third of the tube, the girl immediately grabs the pipe-cleaner and attempts several times to use it to press the handle of the basket against the tube wall and pull it up. The tube is too narrow and the attempts remain unsuccessful. With a hesitant movement, the colorful string is also briefly dangled into the tube before she seems to get distracted (Isen et al., 1987; Subramaniam et al., 2009). Her gaze seems lost for a moment (Segal, 2004; Kohn and Smith, 2009) when suddenly her pupils dilate (Salvi et al., 2020) and a smile appears (van Steenburgh et al., 2012). She expresses a drawn-out and slightly soaring “Aaahhhh!” and immediately grabs the pipe-cleaner, bends a little hook into one of its distal ends, inserts the hooked end of the pipe-cleaner back into the tube, hooks the handle of the basket, pulls the basket over the rim, and claims her reward with determination (Stuyck et al., 2021).

The hook bending paradigm is a so-called ill-structured innovation task in which the path to the solution is missing information about how to get from its start to its goal state (Cutting et al., 2014). Interestingly, children that are seven or older find the entire multistep solution to this problem very suddenly rather than in an incremental way. Notably, the hook bending task has similarly been used to test tool innovation in large brained birds and apes, which show a rather ratchet-like improvement upon solving the task for the first time (rarely failing after first success; Weir, 2002; Bird and Emery, 2009a; Laumer et al., 2017, 2018).

The moment just before the little girl tackles the problem, or what Hermann von Helmholtz referred to as a “happy idea” (Wallas, 1926), may be a familiar sentiment to most of us. Such moments of so-called insight are also a recurringly described (and romanticized) phenomenon in scientific history: Newton and that apple, Archimedes in the bathtub, and Poincaré stepping on the bus; all of them have a common pattern: someone with accumulated experience escapes for a moment from the problem to be solved and suddenly finds themselves surprised (without knowing how or why) with the solution.

## Insight As a Global Phenomenon

Although there are cultural differences in the importance we attribute to insight as a source of creative output (Rudowicz and Yue, 2000; Niu and Sternberg, 2006; Shao et al., 2019), the traditional description of the stages of the creative process is very similar in European psychology (four stage model by Wallas, 1926) and Eastern philosophy (Yoga Sutras; Maduro, 1976; Shao et al., 2019). Insight itself also has an important bearing in Eastern cultures. For example, in Theravada Buddhism, the goal of vipassana meditation is to reach a sudden understanding, abhisamaya (insight), which contrasts with gradually attained understanding (anapurva). Both the description of the phenomenon and the way in which it is achieved, fit with the popular Western notion of insight (Laukkonen and Slagter, 2021).

Although we can have reasonable confidence that insight is a global phenomenon and not a myth specific to western culture (a WEIRD one; Henrich et al., 2010), it still holds many mysteries regarding its mechanisms and function (Shen et al., 2018), as well as its evolution and presence (and level of expression) in other species (Call, 2013).

## Scientific Insight

Given the importance of the subjectively perceived components of insight, the phenomenon is certainly easier to study in humans than in non-human animals, both because of the possibility to report verbally (the subject might describe the suddenness of the solution’s appearance and the emotions involved, but also specific difficulties with aspects of the task, and how close the subject believes he or she is to the solution at any given moment) and the methodology (because of test diversity and the relative ease of applying neuroimaging technology).

A review by Kounios and Beeman (2014) defines insight as any sudden comprehension, realization, or problem solution that involves a reorganization of the elements of a subject’s mental representation of a stimulus, situation, or event to yield a non-obvious or nondominant interpretation. Note, however, that there are various definitions of insight with some considering it as a dynamic process, and others as an end state (Call, 2013; Kounios and Beeman, 2014; Shen et al., 2018). Insight is further frequently linked to a number of traits (such as an impasse or a pleasant feeling of surprise) that may or may not be considered essential to some authors, resulting in variation in the respective definitions (as reviewed in Kounios and Beeman, 2014; and the reason we are using their definition). While neuroscience has been hampered by some inconsistencies in definitions of insight (see Kounios and Beeman, 2014 for examples), experimental evidence (especially due to advances in neuroimaging; e.g., Shen et al., 2018) has helped to guide research along a convergent path (Stuyck et al., 2021), suggesting that innovation achieved through insight-like experiences can be clearly distinguished from other problem solving strategies (van Steenburgh et al., 2012).

Despite the success within neuroscience, the topic of insight and even the use of the term in animal behavior has caused significant theoretical debates in comparative cognition (e.g., Kacelnik, 2009; von Bayern et al., 2009; Emery, 2013). Notably, few animal studies are included the recent literature on human problem solving or neuroscience (Shettleworth, 2012; Call, 2013).

## First Scientific Approximations To Insight

In 1925–1926, Wolgang Köhler and Graham Wallas independently published two books that had long lasting effects on the general perception of problem solving: The Mentality of Apes, by Köhler, and The Art of Thoughts, by Wallas.

Wallas, inspired by the ideas of Hermann von Helmholtz and Henri Poincare, proposed four stages of progression for a creative process (Wallas, 1926). Helmholtz, during a banquet held for his 70th birthday in 1891, revealed how he had reached his best ideas; always after first researching a problem in detail, letting it rest, and seeking a pleasant distraction. This way he was often surprised by a solution in the form of a pleasant experience. Wallas named these stages preparation (investigative stage), incubation (temporally discarding the problem from conscious thought), and illumination (the sudden arrival of a new “happy idea”), to which he added a fourth, the verification of the solution. These four stages have been recurrently used as a framework for studying insight in the psychological literature (Luo and Niki, 2003; Jung-Beeman et al., 2004; Sandkühler and Bhattacharya, 2008; Weisberg, 2013). Although Wallas’ work covers the creative process in rather broad terms, its relevance to the study of insight is remarkable, due to the close proximity and similarity in conceptualization, measures, and processes (Shen et al., 2017, 2018).

**Figure 1:**
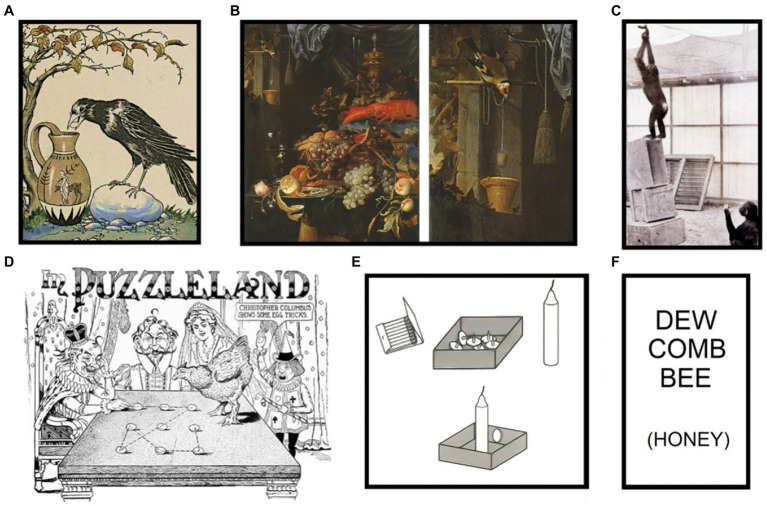
**(A)** The Crow and the Pitcher, illustrated by Milo Winter (1919; Public Domain). Stones must be dropped into water to have access to the liquid, or to a floating object. **(B)** String-pulling; “Still Life with Fruit and a Goldfinch,” Abraham Mignon (1660; Public Domain). Goldfinch’s detail, right side. To have access to the hanging object, the string must be pulled first; as seem in Jacobs and Osvath (2015). **(C)** Three-boxes experiment; “Grande on an insecure construction” The Mentality of Apes, Köhler (1925; CC) To get the banana, the chimpanzees must pile the boxes. **(D)** Early representation of the nine-dot problem; Egg of Columbus, Sam Loyds Cyclopedia of Puzzles (1914; Public Domain). Nine dots, arranged in three parallel lines, must be linked with four connected straight lines. **(E)** Candle problem; Duncker (1945; Public Domain) A candle must be attached to the wall; subjects are given a box of tacks, a candle, and matches. Problem on top, solution, below. **(F)** Compound Remote Associates Test test; developed by Mednick and Mednick (1967). Subjects are given the three words on top and have to find one to link with each one of them (as the one in brackets). All Public Domain and Creative Commons (CC) images can be found in Wikimedia Commons.

Almost at the same time, Wolfgang Köhler, one of the pioneers of Gestalt psychology, introduced the term insight into comparative psychology (although this way of problem solving was already described before him in non-human animals; Turner, 1909; Köhler, 1925; Weisberg, 2006; Galpayage Dona and Chittka, 2020). Gestalt psychologists proposed that insight depends on different mechanisms to trial and error learning, which, according to Thorndike (1911), was the only way in which animals could solve problems (Köhler, 1925; Koffka, 1935; Duncker, 1945; Wertheimer, 1959). Köhler worked for years at the Casa Amarilla in Tenerife (Canary Islands, Spain) with seven chimpanzees, testing them in experiments where they had to find unusual methods to reach food (see Figure 1). In those experiments, Köhler found problem solving strategies that did not seem compatible with classical associative learning routines: After an unsuccessful period of trial and error, in which the chimpanzees used familiar strategies, they stopped trying. Nevertheless, after a while some of them returned with a completely different and, this time, immediately successful strategy. After their first success, the animals could immediately retrieve the correct sequence of steps on the following occasions when they faced the same problem. Köhler, at the time, described these strategies as cognitive trial and error and insight, rather than associative processes.

Other Gestalt psychologists adapted Köhler’s problem solving methodology to study insight in humans. Duncker (1945), for example, designed situations in which everyday objects had to be used in unusual ways to solve a task (e.g., the candle problem, see Figure 1; Duncker, 1945). Notably, if he asked the subjects to use these objects in their usual way before the test, the success rate was reduced. Duncker and other Gestalt psychologists (e.g., Maier, 1930; Luchins, 1942; Scheerer, 1963) concluded that the repeated application of incorrectly selected knowledge could prevent the deep conceptual understanding necessary to achieve insight. This phenomenon is now known as functional fixedness (Duncker, 1945).

It was, however, the British ornithologist W. H. Thorpe who coined in his book Learning and Instinct in Animals (1956) the most prevalent definition of insight in psychology today; “*the sudden production of a new adaptive response not arrived at by trial behaviour or the solution of a problem by the sudden adaptive reorganization of experience*.” We will later explain how an over-emphasis on the absence of trial and error learning, and a lack of attention to the “reorganization of experience,” may have affected the interpretation of insight in comparative cognition.

## Our Current Understanding of Insight

Insight is often conceptualized as a process in which a subject has a sudden realization of how to solve a novel problem (Schooler et al., 1995; Sheth et al., 2009). Thereby specific elements of a subject’s mental representation of various stimuli, situations, or events are reorganized to yield a nonobvious or nondominant interpretation (Kounios and Beeman, 2014). Insight is associated with a number of characteristic phases that set it apart from other mental processes employed in problem solving, such as a distinctive subjective momentary experience of surprise and delight, the “aha” or “eureka” moment (Bowden et al., 2005).

Neuroscience typically contrasts insight with analytical reasoning within problem solving. A directly perceivable difference between the two seems to be a more or less gradual progress toward a solution in analytical thinking (Smith and Kounios, 1996), while individuals are abruptly surprised by the latter during an insightful solution (Metcalfe and Wiebe, 1987). Thus, insight is believed to depend by a large degree (but not completely) on unconscious mental processing, as we will see in the next sections (Sandkühler and Bhattacharya, 2008; Shen et al., 2013, 2018; Weisberg, 2013).

### Convergent Insight Process Theories

The main theoretical proposals to explain insight largely differ with regards to the amount of conscious processing they describe involved in an insightful event. For example, approaches, such as the representational change theory (also called the redistribution theory; (Ohlsson, 1992, 2011; Knoblich et al., 1999), advocate a completely unconscious redistribution of information (Knoblich et al., 1999; Ohlsson, 2011), whereas the progress monitoring theory (or criterion for satisfactory progress theory; MacGregor et al., 2001; Chu et al., 2007) proposes insight through a conscious process: searching consciously among a pool of possible solutions during which wrongful presumptions are dropped in favor of a working solution.

In an attempt to find a bridge between the strengths of both previous theories, Weisberg proposed an integrated theory of insight comprising several phases: the individual would first attempt to find a solution by using strategies based on long-term memory; if this fails, the subject would use rules of thumb or more complex heuristics to acquire information about the problem before re-confronting its long-term memory; then, a conscious solution *via* a restructuring of old and new information may thereby be achieved; and if the process reaches an impasse and new information is no longer acquired, an unconscious restructuration of knowledge would take place (Weisberg, 2015). Interestingly, the four stages of Weisberg's (2015) proposal bear some parallels to those suggested by Wallas in the mid twentieth century (Wallas, 1926). “Preparation” would comprise the first three phases of the integrated insight theory, while “incubation” and “illumination” could be interpreted as part of the fourth, where insight is achieved through an unconscious process (see above, section four, to find Wallas’ proposal).

### Fixation and Impasse

The fixation and impasse (the repetition of incorrect strategies, and the following temporary withdrawal of action), as already described by Duncker (1945), are likely the result of an inappropriate knowledge base (Wiley, 1998) or incomplete heuristics (Knoblich et al., 1999, 2001). Knoblich et al. (1999) found that expertise in algebra can negatively affect insightful arithmetic problem solving. Similarly, great apes have trouble innovating a solution to a problem when the tools or objects at their disposal were previously used in a different way (Hanus et al., 2011; Ebel et al., 2020). Such “functional fixedness” may be one of the factors responsible for the fixation leading to an impasse.

It is important to highlight at this point that there are no insight problems but only insight solutions: any problem solved by insight could also be solved analytically (van Steenburgh et al., 2012), and that an impasse (although common) is not required for insight to occur (MacGregor et al., 2001; Ormerod et al., 2002; Kounios and Beeman, 2014). However, the design of a problem is highly important as it determines the nature of its solution/s. Experimental subjects in classical insight challenges, such as Duncker’s candle problem (e.g., Duncker, 1945; Knoblich et al., 2001; Huang, 2017), often encounter an impasse prior to the solution. This is much less common in so-called CRAT-based challenges (a specific type of word puzzle, see Figure 1; e.g., Cranford and Moss, 2012; Webb et al., 2019) even if they are also solved by insight. This could be because classical tests often have misleading structures and/or contain elements that may provoke functional fixedness (Duncker, 1945; Hanus et al., 2011; Stuyck et al., 2021). Nevertheless, the scientific approach for detecting an impasse may also be problematic (Stuyck et al., 2021): Studies that found no impasse before insightful solutions mostly relied on verbal reports (e.g., Webb et al., 2019), while when other methods were used an impasse was more likely to be detected (e.g., eye tracking, Huang, 2017; neurophysiological measurements, Shen et al., 2018).

### Incubation/Restructuring and Illumination

An impasse is usually followed by an incubation/restructuring stage, which is suspected to constitute the insight’s core (Wallas, 1926; Sandkühler and Bhattacharya, 2008; Sio and Ormerod, 2009; Cranford and Moss, 2012; Weisberg, 2013). Although restructuring can of course be done consciously (Weisberg, 2015), it may also happen at a time during which a subject consciously withdraws from the problem at hand (van Steenburgh et al., 2012; Kounios and Beeman, 2014; Shen et al., 2018). We know that insight-like responses improve when participants take a break after reaching an impasse (or when the task is simply removed from their sight; Kohn and Smith, 2009), regardless of the duration of the break, and particularly when the break is occupied with a different, cognitively demanding task; Segal, 2004).

Human neuroimaging and electrophysiology-based studies suggest a significant function of the prefrontal cortex in the process of overcoming impasse to reach incubation (e.g., Qiu et al., 2010; Zhao et al., 2013; Seyed-Allaei et al., 2017; Shen et al., 2018). The right inferior frontal gyrus plays a role in evaluating possible solutions while the left gyrus seems to control the suppression of inappropriate mental sets or dominantly activated associations (e.g., Jung-Beeman et al., 2004; Shen et al., 2013, 2018; Wu et al., 2015). This corresponds with studies reporting brain asymmetries in insight tests. Studies using insight and priming with word hints (where the left hemisphere typically has an advantage; van Steenburgh et al., 2012), the left visual field (right hemisphere) has shown a strong advantage over the right, with primed participants finding more solutions faster (Bowden and Beeman, 1998; Beeman and Bowden, 2000).

Studies based on event-related potentials have so far been able to identify two distinct cognitive processes involved in achieving an insightful event: the breaking down of the impasse (allowing incubation/restructuring) and the formation of new associations prior to the solution (Luo and Niki, 2003; Luo et al., 2011; Zhao et al., 2013; Shen et al., 2018; it is also described as the enlightenment stage by Wallas, 1926).

Associations that will result in a solution can take different routes; once strong yet incorrect associations can be overcome, weaker yet correct association can be detected (Shen et al., 2018). Interestingly, the latter is facilitated by a positive emotional state (Isen et al., 1987; Subramaniam et al., 2009; van Steenburgh et al., 2012). In humans, a positive emotional state at the start of testing is associated with increased activity in the anterior cingulate cortex (which is related to monitoring cognitive conflict; Carter et al., 2000) and an increase in insightful solutions (Subramaniam et al., 2009).

While neurobiology and cognitive psychology embrace insightful solutions achieved by associations learned in the past, comparative cognition tends to exclude associative learning from its notion of insight, which is a misconception as insight can occur through distant or weak associations (Shettleworth, 2012; Call, 2013). In comparative cognition, insight has occasionally been used as a default explanation upon failing to detect the typical gradual process of associative learning.

A candidate for explaining how we can learn non-obvious associations is latent learning (Tolman and Honzik, 1930; Tolman, 1948). The nervous system can register associations without the need for positive reinforcement (such as those that can be acquired through random exploration). These associations remain latent and are candidates for insightful solutions (Thorpe, 1956). Latent associations, being weak, can be adjusted more flexibly if required (Call, 2013). In contrast, strong associations can result in functional fixedness where a previous solution prevents the innovation of a new solution (e.g., humans, Duncker, 1945; great apes, Ebel et al., 2020).

However, the path toward a solution can be achieved by other mechanisms. The free energy principle [the basis of Predictive Processing Theory (PPT), e.g., Hohwy and Seth, 2020; Francken et al., 2021] predicts that all sentient beings minimize uncertainty for energetic reasons (Friston, 2003). According to PPT, all interaction with the environment involves constant amendment between perceptual input and the internal models (Friston et al., 2016a). When the flow of input stops during an impasse, models continue to be optimized without the agent consciously perceiving it. This has been called fact-free learning or model selection and reduction (model selection, Aragones et al., 2005; model reduction, Friston et al., 2016b). In the absence of new data, the only way we can optimize our generative models is by making them simpler (Friston et al., 2017).

Model reduction is a similar process to that described in the N-REM phase of sleep, where redundant connections between neurons are eliminated (Tononi and Cirelli, 2006) and models are reduced in complexity in the absence of new sensory input (Friston et al., 2017).

Model reduction occurs neither only during sleep, nor only in humans. Rats that move away from exploratory or spatial foraging behavior, and enter short periods of rest, have been found to have hippocampal activity similar to what we would expect in models undergoing insight-compatible changes (Gupta et al., 2010; Pezzulo et al., 2014; Friston et al., 2017). Internally generated sequences (sequences of multi-neuron firing activity that do not reflect an ongoing behavioral sequence) seem to be able to restructure models, not only consolidating memory but also exploring potential solutions (Pezzulo et al., 2014).

### The Eureka Experience

A popular event related to insight is the so-called “aha” moment, a subjective experience of surprise and delight accompanied by sudden solutions (Bowden et al., 2005; Sandkühler and Bhattacharya, 2008; Weisberg, 2013; Shen et al., 2017). This pleasant experience is probably one of the reasons why insight responses are associated with positive emotions versus analytical solutions that are negatively perceived (Shen et al., 2016, 2017; Webb et al., 2016, 2019). This may also contribute to a better memorization and a higher success rate of insightful responses (e.g., Danek et al., 2013; Webb et al., 2016; Salvi et al., 2020; Stuyck et al., 2021).

Notably, insight does not necessarily require this “aha” experience. In verbal tests, insight lacking major emotional changes has been reported (Kounios and Beeman, 2014). This may be the reason why CRAT tests do not elicit a perceivable impasse experience (Stuyck et al., 2021). Nevertheless, the impasse may be an important contributing factor to the surprise element of the insight revelation as it fosters the perception of a metacognitive error in which we solve a problem faster than expected (Dubey et al., 2021).

The subpersonal nature of model reduction (that is, there is no explicit inner model, hence no conscious experience of the reduction process) could explain why the agent becomes aware at the precise instance of a new association, and not before (Metcalfe and Wiebe, 1987; Friston et al., 2017; Shen et al., 2018). Another proposed explanation for the relation of insight with consciousness is the asymmetrical involvement of both hemispheres and the important role of the right hemisphere in key parts of the process (see split brain perception studies, e.g., Gazzaniga, 1998; van Steenburgh et al., 2012). Furthermore, the conscious perception of the solution is plausible considering the close relationship between associative learning and consciousness (Ginsburg and Jablonka, 2007, 2019) and the essential role of consciousness for the former to occur (e.g., Baars et al., 2013; Meuwese et al., 2013; Weidemann et al., 2016).

## Non-Human Animals, Problems, and Solutions

Comparative cognition has attempted to tackle the presence of insight in animals by rating the speed of their performance on technical problem or their ability to transfer information from one task to another (Seed and Boogert, 2013).

One issue with this may be that, as mentioned earlier, there are no insight problems, only insight solutions; a problem designed to be solved by insight can also be solved by other processes (van Steenburgh et al., 2012). Epstein et al. (1984) tried to highlight this issue in a popular paper which showed that pigeons solved seemingly complex problems spontaneously by “chaining” blocks of previously learned information.

Neuroscience’s results and advances have been able to compensate a lack of theoretical consistency regarding insight. Cognitive research on animal insight, on the other hand, has been limited to the creativity of experimental designs, with no apparent chance of ending long-running debates stemming from two opposing schools of thought, cognitive psychology and behaviorism, “romantics” against “killjoys” (Shettleworth, 2010, 2012; Call, 2013; Starzak and Gray, 2021). While we believe that the progress of comparative cognition feeds (as a dissipative structure) on the continued conflict between the two positions, the lack of experimental progress has kept these discussions in an impasse (e.g., Heinrich, 1995; Kacelnik, 2009; Chittka et al., 2012; Taylor et al., 2012; Emery, 2013; Starzak and Gray, 2021).

Today we know that insight is a measurable phenomenon with a physiological basis that is beginning to be revealed (Shen et al., 2018). Moreover, it makes little sense to set the phenomenon apart from associative learning and experience (Shettleworth, 2010, 2012; Hanus et al., 2011; Call, 2013; Shen et al., 2018; Ebel et al., 2020). Insight does not mean developing *de novo* behaviors to solve a problem, but to find a solution by restructuring the problem, even if the agent reorganizes old experiences to apply them to a novel context.

Although insight involves making the nonobvious seem obvious, and even tends to correlate with a higher success rate at problem solving (higher successful rate, Salvi et al., 2016; Webb et al., 2016; but see, Stuyck et al., 2021), a successful restructuring does not necessarily imply a correct conceptualization of the full nature of the problem, and an answer obtained by insight need not necessarily be correct (Kounios and Beeman, 2014). Just as a feeling of understanding does not equate to a true understanding of the problem, we must thus be careful in equating insight with understanding or suggesting that one predicts the other.

Insight may exist in animals outside humans and could even be relatively widespread in nature (e.g., Shettleworth, 2012; Pezzulo et al., 2014). Yet to proficiently tackle the phenomenon in non-verbal species is an unsolved problem in comparative cognition.

While rodent studies suggest that insight does not require sophisticated cognition, the role of the prefrontal cortex in important insight stages may suggest insightful solutions are more likely to emerge in species that have highly developed and functionally equivalent brain regions (Shettleworth, 2010, 2012; Call, 2013; Olkowicz et al., 2016; Shen et al., 2018).

Methodologies, such as the priming of different brain hemispheres, related to insight (which function similarly in non-human primates as in humans) as well as new technologies in animal eye tracking open the door to technically challenging targeted studies in species other than our own (Krupenye et al., 2016; Shen et al., 2018; Völter et al., 2020; Ben-Haim et al., 2021).

The crucial role of subjective experience in insight, as well as the traditional reliance on verbal reports in a large number of studies, makes it tempting to conclude that the study of insight is inaccessible in non-human animals. Nonetheless, other signatures of insight do exist (e.g., Kounios and Beeman, 2014). Apart from EEG and fMRI studies, evidence of human insight stems also from eye tracking studies (e.g., Salvi, 2013; Salvi et al., 2016; Huang, 2017), grip strength (Laukkonen et al., 2021), heart rate (Hill and Kemp, 2018), pupil dilation, and eye movement (with pupil dilation happening only just prior to an insightful event, and an increase in microsaccade rate coinciding with analytic responses; Salvi et al., 2020). Moreover, it has been shown repeatably that agents do not even necessarily need to solve the problem. A promising approach could be to confront an animal with a problem and then, after a period unsuccessful interaction, to suddenly show the solution and record the response (e.g., Kizilirmak et al., 2016; Webb et al., 2019).

Even the “aha” moment itself might be accessible to study in non-verbal subjects, given the expected physiological emotional response that follows it. We know that many animals show an emotional response while learning how to solve tasks (independent from the presence of a reward; e.g., cows, Hagen and Broom, 2004; goats, Langbein et al., 2004; horses, Mengoli et al., 2014; dogs, McGowan et al., 2014; dolphins, Clark et al., 2013). Studying insight through the presentation of a solution would thus require both a behavioral analysis (as in traditional contrafreeloading tests or yoked experimental designs; e.g., Hagen and Broom, 2004; Rosenberger et al., 2020) as well as a physiological one. Artificially altering the transparency of the path toward the solution, and altering the time spent at an apparent impasse, may allow us to predict and modify the intensity of the respective physiological (as it would be an increased heart rate; Hill and Kemp, 2018) and behavioral responses (e.g., in dogs, we would predict pupil dilation, tail wagging, and increased general activity; McGowan et al., 2014; Webb et al., 2019; Salvi et al., 2020).

## Conclusion

Insight is a measurable phenomenon in humans, and the mechanisms by which it occurs may well be accessible to species other than our own. Thanks to recent progress in neuroscience and human psychology, we are beginning to clarify the (in some cases subtle) differences that distinguish insight problem solving from other processes. Comparative cognition, however, has so far been limited in its approach. Performance-based setups using technical problems in both birds and mammals have produced highly interesting and suggestive, yet, ambivalent evidence on animal insight (e.g., Heinrich, 1995; Mendes et al., 2007; Bird and Emery, 2009a,b; Laumer et al., 2017, 2018; von Bayern et al., 2018). We are optimistic that accomplishments in neuroscience and human psychology over the past decade can be incorporated into and inspire future comparative cognition studies in their ongoing quest to learn about the capacity for insight in species other than our own.

## Author Contributions

AO-M wrote the first draft. AO-M and AA finished the manuscript. All authors contributed to the article and approved the submitted version.

## Funding

The authors are funded by the WWTF Project CS18-023 and START project Y 01309 by the Austrian Science Fund (FWF) to AA.

## Conflict of Interest

The authors declare that the research was conducted in the absence of any commercial or financial relationships that could be construed as a potential conflict of interest.

## Publisher’s Note

All claims expressed in this article are solely those of the authors and do not necessarily represent those of their affiliated organizations, or those of the publisher, the editors and the reviewers. Any product that may be evaluated in this article, or claim that may be made by its manufacturer, is not guaranteed or endorsed by the publisher.
